# Factors influencing how informal caregivers of people with multiple sclerosis access and use a curated intervention website: Analysis from an RCT

**DOI:** 10.1177/20552076241228403

**Published:** 2024-02-08

**Authors:** Tanya Packer, Nichole Austin, Michelle Lehman, Sara L Douglas, Matthew Plow

**Affiliations:** 1 Schools of Health Administration and Occupational Therapy, Dalhousie University, Halifax, Nova Scotia, Canada; 2 School of Health Administration, Dalhousie University, Halifax, Nova Scotia, Canada; 3 Frances Payne Bolton School of Nursing, 2546 Case Western Reserve University, Cleveland, OH, USA

**Keywords:** Informal caregiver, e-health, virtual care, multiple sclerosis, caregiver burden

## Abstract

**Objective:**

Healthcare consumers and providers are increasingly turning to digital solutions, such as curated websites. Knowing who accesses/benefits from these may improve design and development. This study investigated website usage of informal caregivers of people with multiple sclerosis and shifts in outcome plausibly associated with usage.

**Methods:**

Secondary analysis of data from a randomized clinical trial of 148 caregivers compared effectiveness of a website + tele-coaching to a website only intervention for caregivers. Groupwise differences in means/proportions were tested using t-tests and chi-square. Modified Poisson regression with a robust variance estimator and ordinal logistic regression tested the relationship between group and likelihood of website log-in. Ordinal logistic regression models examined whether caregiver characteristics were associated with website use. Generalized estimating equations (GEE) with an autoregressive correlation structure modeled the relationship between website usage and outcomes.

**Results:**

Females were more likely to access the website than males (60% vs. 43%; p = 0.05). Though not statistically significant, a possible association (POR: .85, 95% CI: .69, 1.03) between caregiver burden and website access emerged; caregivers experiencing highest levels of burden appeared less likely to engage. Usage patterns differed by treatment arm: the website-only group accessed the *Caring for yourself* topic significantly more (61.67% vs. 38.33%: p = .04) with similar, but insignificant, trends for other topics.

**Conclusions:**

Clinicians can be confident referring females with moderate levels of burden to website-based interventions. By contrast, male caregivers and those experiencing high levels of burden may be less likely to access these resources, pointing to the need for alternative interventions.

**Trial Registration:**

Clinicaltrials.gov, registration number: NCT0466208.

## Introduction

Advances in, and availability of, technology, coupled with the exponential increase in virtual care during the COVID-19 pandemic, means patients, families, and providers will continue to turn to digital options as an alternative, enhancement, or complement to face-to-face healthcare delivery.^1–3^ For patients and informal caregivers, virtual care reduces barriers to travel, wait times, and scheduling.^4^ Unpaid caregivers of people with multiple sclerosis (MS) spend between 4.6 to 12 h per day providing care,^5^ compressing time for all other activities. Strategies to relieve burden and save time are critical.

In the United States, virtual care, defined as any remote communication or information technology used between patients and health providers (e.g., phone calls, text message, emails, videoconferencing), increased close to 30 times between September 2019 and September 2020, with many clients becoming first-time users.^6^ Studies show that 80% of clients are satisfied with virtual care and 75% wish it to continue.^7–9^ The use for both routine care^10,11^ and structured interventions (coaching, rehabilitation, and self-management)^12,13^ is likely to grow.

During COVID-19 many structured, face-to-face programs were adapted for virtual delivery. While some rely completely on digital or technical solutions such as passive or interactive websites, others are deployed via telephone or videoconference.^14^ Hybrid models also exist with interventions often accompanied by printed manuals, websites, asynchronous online components, virtual social networks or discussion boards and/or monitoring and tracking systems (see e.g., Lynch et al.,^15^ and Kelleher et al.^16^). Interventions to support informal caregivers are no exception, often taking the form of curated websites with^16^ and/or without augmentation.^17^ Evidence of effectiveness that began emerging prior to COVID-19^18,19^ is now growing.

The development of websites and digital interventions, however, is costly and time-consuming. Website appearance and navigation, content and messaging, and intervention burden (time and cognitive) must be considered.^20^ Evidence has also raised practical issues related to access, preference, and retention. Pre-COVID studies suggest high attrition rates. For example, a review of 19 studies of web-based interventions with minimal therapist contact published between 1990 and 2009 found drop-out rates up to 24%, with many participants dropping out before even accessing the website.^21^ Because of high dropout rates, Eyesnbach et al. (2005) argued the need for a theory to explain the “Science of Attrition” in eHealth trials.^22^ A 2020 report, however, found that participants in the videoconference arm of a pain intervention were significantly more likely than those in the in-person arm to complete all sessions (83% vs. 65%; *P* = 0.006).^23^ Similarly, in 2021 Lock^24^ found that both a self-guided online and a videoconference family intervention for parents of a child with anorexia were feasible, had similar recruitment and retention rates, and had equal acceptability to parents.

Most studies report higher participation rates for females versus males both in caregiver studies and non-caregiver studies. Characteristics associated with attrition, however, are less consistent. For example, Pedersen et al. reported that participants who were female and over the age of 75 were more likely to drop out of lifestyle interventions.^25^ Conversely, Melville, Casey, and Kavanagh found males and younger participants had higher attrition rates from internet-based treatments for psychological disorders.^21^ Less formal education, lower income, and socioeconomic status are more consistently associated with high attrition.^21,26^

As healthcare becomes increasingly virtual, knowing who accesses and benefits from curated websites will inform the design and development of interventions for patients and their informal caregivers.^3^ Data from a two-arm, randomized clinical trial of 150 informal caregivers of people with multiple sclerosis (MS Caregivers) provided an opportunity to investigate website usage.^27^ The RCT compared effectiveness of two interventions: a website + tele-coaching psycho-educational intervention, and a website only intervention, to reduce caregiver stress, anxiety, depression, distress, and burden. In the present analysis, our overarching goals were to better understand website usage patterns, and to identify any detectable shifts in outcome measures plausibly associated with usage across both treatment arms of the study.

## Methods

This analysis of existing data sought to answer three specific questions:

RQ1. Among MS Caregivers, who was most likely to access a curated, password protected website?
RQ2. Is there a relationship between caregivers’ baseline burden/depression/anxiety/stress measures and the website pages they access?
RQ3. Does website use influence outcomes at follow-up?

While both intervention groups had access to the same curated, password protected website, only the website + tele-coaching arm received four individual coaching sessions with a social worker.^27^ Ethics approval was received from both the Case Western Reserve University Institutional Review Board and the Dalhousie University Research Ethics Board. All participants gave consent prior to participation via an electronic consent form sent by REDCap for online signature.

### Recruitment

Recruitment for the RCT took place in the United States from March 2021 to August 2021 with data collection ending in January 2022 via a list of people with MS who gave consent to be contacted for future research (PCORI Grant No. MS-1610-37015), via an email sent by the National Multiple Sclerosis Society to all caregivers in their database, or via an IRB-approved flier posted on Facebook. Caregivers were any close friend or family member providing any type of support (e.g., physical, emotional, administrative—such as paying bills), i.e., not a professional or paid for their services. Included caregivers (a) self-identified as an adult caregiver (18 years or older), (b) gave informed consent, (c) identified English as their primary language, and (d) were able to access the internet. No specific exclusion criteria were applied. All participants were eligible for inclusion in this analysis. Prior to the RCT, a mixed effect model power calculation, assuming a correlation of 0.5 among the 3 repeated measurements, a small Cohen's d of 0.35 and 20% attrition rate, estimated a need for 75 participants per treatment group.^27^ These estimates are consistent with previous studies of psychoeducational interventions with caregivers.^28,29^ For a more detailed description of the recruitment, allocation and completion and the trial flow diagram please see Douglas et al.^28^

### Interventions

Both interventions were psycho-educational in design.^27^ The website, accessed by participants in both interventions, was specifically curated by the research team and housed on a secure server meeting privacy requirement of both the US Health Insurance Portability and Accountability Act (HIPAA) and the Canadian Personal Information Protection and Electronic Document Act (PIPEDA). Participants were assigned unique password logins, used to track website use. No sociodemographic information was collected or stored on the website.

Modeled on a successful website for caregivers of patients with cancer,^28^ the website houses text information, web links, and video scenarios in six topic areas: (a) information about MS, (b) obtaining reliable online information about MS, (c) caring for your loved one with MS, 4) COVID-19 and MS, 5) caring for yourself, and 6) planning and decision-making. Video scenarios were co-created with MS Caregivers who were part of the study's advisory group. Both groups were introduced to the website by the Project Director who emailed them the logon information and their unique password. The email received by the control group included a sentence identifying key areas found within the website. Both groups also received this information when consent was obtained. Participants could access the website as many or as few times as they wished.

The website + coaching group also received four personalized coaching sessions (over 6 weeks) with a trained and licensed independent social worker guided by a facilitator manual and delivered via videoconference or telephone, per participant preference. Individualized information and support, based on individual assessments, discussions, and skill building exercises, was provided in areas consistent with the content on the website: Session 1, identifying needs of caregivers for information and support; Session 2, strategies for caring for a loved one with MS; Session 3, caring for yourself; and Session 4, planning and decision-making for the future. While the coaching did not specifically encourage website usage, the first session included an informational component that referred to the website and discussed the type and location of information available.

### Measures

Consent and self-report survey data were collected online via the Research Electronic Data Capture (REDCap) platform. Sociodemographic characteristics of MS Caregivers and their family/friend with MS (as reported by MS Caregiver), care-related duties and mental health data were collected at baseline. Follow-up outcome measures were collected 6 weeks after baseline (following the intervention period) and again six weeks later. Standardized measures were used to measure caregiver depression, anxiety, and stress (Depression, Anxiety and Stress Scale, DASS^30^), caregiver distress (NCCN^®^ Distress Thermometer Tool^31^), and caregiver burden (Zarit Burden Interview Short^32^). All have been shown to be psychometrically sound.^33^

Website usage was captured dichotomously (did/did not access), categorically (no usage, low, medium, high) and as a % of content accessed (education elements = page visits, video views, links accessed). Dichotomous use was based on whether a participant ever logged into the website, regardless of their subsequent web use/navigation patterns. Categorical usage was derived by dividing web usage into four groups based on percentiles: no usage (no interaction with educational elements), low usage (interaction with ≤6 educational elements), medium usage (interaction with >6 but <15 educational elements), and high usage (interaction with >15 educational elements).

### Statistical analyses

To assess which MS Caregivers were more likely to access the curated website (**RQ1**), we compared baseline sociodemographic characteristics of individuals who did (vs. did not) log into the website; groupwise differences in means/proportions were assessed via t-tests and chi-square tests, respectively (α=.05). We assessed whether caregivers in the website + coaching intervention group were more likely to log in than those in the web-only intervention group (as group assignment was randomly allocated, no additional covariates were included in these models). The relationship between intervention group assignment and the binary likelihood of logging in (vs. never logging in), as well at the relationship between group assignment and categorical web usage (no usage, low, medium, high) were next evaluated. For the binary (did/did not access) login measure, modified Poisson regression with a robust variance estimator was chosen. A common alternative to logistic regression for binary outcomes, it has the added advantage of directly estimating risk ratios (RRs) instead of odds ratios (ORs).^34^ A risk ratio of 1 was considered indicative of a null impact with risk ratios greater than 1 reflecting a higher likelihood of logging in. This difference is considered statistically significant if the accompanying confidence interval does not include 1, the null value.

To test the categorical measure (no, low, medium, high usage), ordinal logistic regression was used. Both models/outcomes used marginal postestimation to re-express associations on the probability scale to assist interpretability. A separate series of ordinal logistic regression models examined whether caregiver characteristics (see below) were associated with higher levels of categorical web use.

To assess whether caregiver baseline levels of depression, anxiety, and stress (DASS), distress (Distress Thermometer) and caregiver burden (Zarit Burden Interview) were related to usage patterns/website pages accessed (**RQ2**), we restricted our sample to users who had accessed the website. Prior to analysis, missing data for DASS items were imputed using the multiple imputation procedure in SPSS. Depth of these users’ engagement with each of the six topics/pages within the website (measured as the percentage of content accessed), as well as the relationship between caregivers’ baseline measures and website page access (to better understand, for example, if users with high baseline burden scores were more or less likely to engage with certain elements of the website), were examined.

Finally, we modeled the relationship between categorical web usage and outcomes at follow-up on four outcome measures (**RQ3**): the three DASS sub-scores (depression, anxiety, and stress) and the Distress Thermometer. MS Caregivers were randomly assigned to the intervention group, not to web usage category (no, low, medium, high use). Their level of website engagement was, therefore, self-determined, meaning control for confounding variables was necessary for this analysis. Covariate selection was guided both by lasso, an automated variable selection method that identifies potentially important predictors, and substantive knowledge/beliefs of factors believed to influence MS Caregivers’ web usage and their subsequent mental health outcomes (e.g., caregivers’ gender, hours of care per week, time since diagnosis, treatment group assignment, baseline outcome measures^35^). We also included an intervention group × usage category interaction term to allow the effect of website usage to vary by group assignment.

To account for within-subject correlation over time (participants’ outcome measures at follow-up would be influenced by their baseline scores), we used generalized estimating equations (GEE) with an autoregressive correlation structure. GEE is an approach to fitting generalized linear models on repeated measures that accounts for within-subject/within-group correlation. Results are interpretable as the population-averaged effect of the intervention—in our case, the extent of web use—on the outcome measures.

## Results

### RQ1. Who accesses a curated, password protected website?

**Factors associated with logging in.** A total of 151 subjects were randomized to the two arms of the intervention. We excluded one individual with a gender listed as “Other”, and two individuals with faulty website credentials (rendering their web usage invisible), bringing our analytical sample to 148. Female MS Caregivers composed approximately 50% of participants (Table 1). Given that approximately 75% of people with MS in the United States are women,^34^ and that caregivers are frequently spouses, a higher proportion of male caregivers might have been expected. MS caregiver characteristics were generally balanced among participants who did (vs. did not) log into the website, except for gender, with female caregivers significantly (60% vs. 43%; p = 0.05) more likely to log in than male caregivers. Baseline measures were comparable between treatment groups.

**Table 1.** Baseline characteristics by group allocation and future web use.

	By treatment assignment			By login category		
	Web only (n = 76)	Web + coaching (n = 72)	*p*-value^a^	Logged in (n = 94)	Did not log in (n = 54)	*p*-value^a^
MS Caregiver characteristics^b^						
Age (years)	53.20 (14.13)	52.17 (13.62)	0.66	53.01 (13.44)	52.17 (14.64)	0.73
Female: %	52.63%	54.17%	0.85	59.57%	42.59%	0.05
Rural	46.67%	40.58%	0.46	40.86%	49.02%	0.35
%White	86.67%	91.67%	0.33	87.10%	92.59%	0.3
Caring for spouse (vs. other)	81.58%	76.39%	0.44	78.72%	79.63%	0.9
Employed						
Full-time	56.58%	51.39%	0.49	55.32%	51.85%	0.56
Part-time	7.89%	15.28%		11.70%	11.11%	
Retired	23.68%	25.00%		25.53%	22.22%	
Not employed	11.84%	8.33%		7.45%	14.81%	
Income						
<=$20,000	6.67%	12.86%	0.64	7.53%	13.46%	0.64
$20,001-$49,999	24.00%	22.86%		23.66%	23.08%	
$50,000-$99,999	36.00%	31.43%		33.33%	34.62%	
>=$100,000	33.33%	32.86%		35.48%	28.85%	
CP hours per week						
<10	27.63%	31.94%	0.94	30.85%	27.78%	0.52
10–20	22.37%	18.06%		21.28%	18.52%	
21–30	9.21%	9.72%		6.38%	14.81%	
31–40	6.58%	8.33%		8.51%	5.56%	
>40	32.21%	31.94%		32.98%	33.33%	
Distress Thermometer (range: 0–10)	3.97 (2.71)	3.92 (2.65)	0.45	3.96 (2.57)	3.92 (2.87)	0.95
Zarit Caregiver Burden (range: 0–16)	6.64 (3.37)	7.44 (3.54)	0.16	7.12 (3.47)	6.87 (3.46)	0.68
DASS-Composite^c^ (range: 0–115)	23.96 (22.85)	26.19 (22.29)	0.55	23.86 (20.27)	27.11 (26.08)	0.4
DASS-Depression^c^ (range: 0–41)	7.28 (8.28)	8.11 (8.41)	0.55	7.49 (7.70)	8.01 (9.39)	0.72
DASS-Anxiety^c^ (range: 0–36)	4.89 (6.58)	4.69 (6.08)	0.85	5.84 (7.60)	4.19 (5.42)	0.13
DASS-Stress^c^ (range: 0–40)	11.79 (9.34)	13.40 (9.51)	0.3	12.17 (9.02)	13.27 (10.15)	0.5
Person with MS characteristics						
Age	50.59 (13.18)	50.10 (11.89)	0.81	51.77 (12.70)	47.89 (11.96)	0.07
Time since dx (years)	13.07 (10.56)	12.38 (9.76)	0.69	13.05 (10.22)	12.17 (10.09)	0.61
Gender: F (%)	67.11%	63.89%	0.68	60.64%	74.07%	0.1
MS type						0.69
Relapsing	54.17%	65.71%	0.16	61.11%	57.69%	
Progressive	45.83%	34.29%		38.89%	42.31%	
Treatment assignment						
Web-only	-	-	-	53.19%	48.15%	0.56
Web + coaching	-	-	-	46.81%	51.85%	

^a^ Based on *t*-tests for continuous variables and chi-square tests for categorical.

^b^ Presented as either mean (standard deviation) or % depending on the variable type.

^c^ Imputed values.

**Figure 1.**
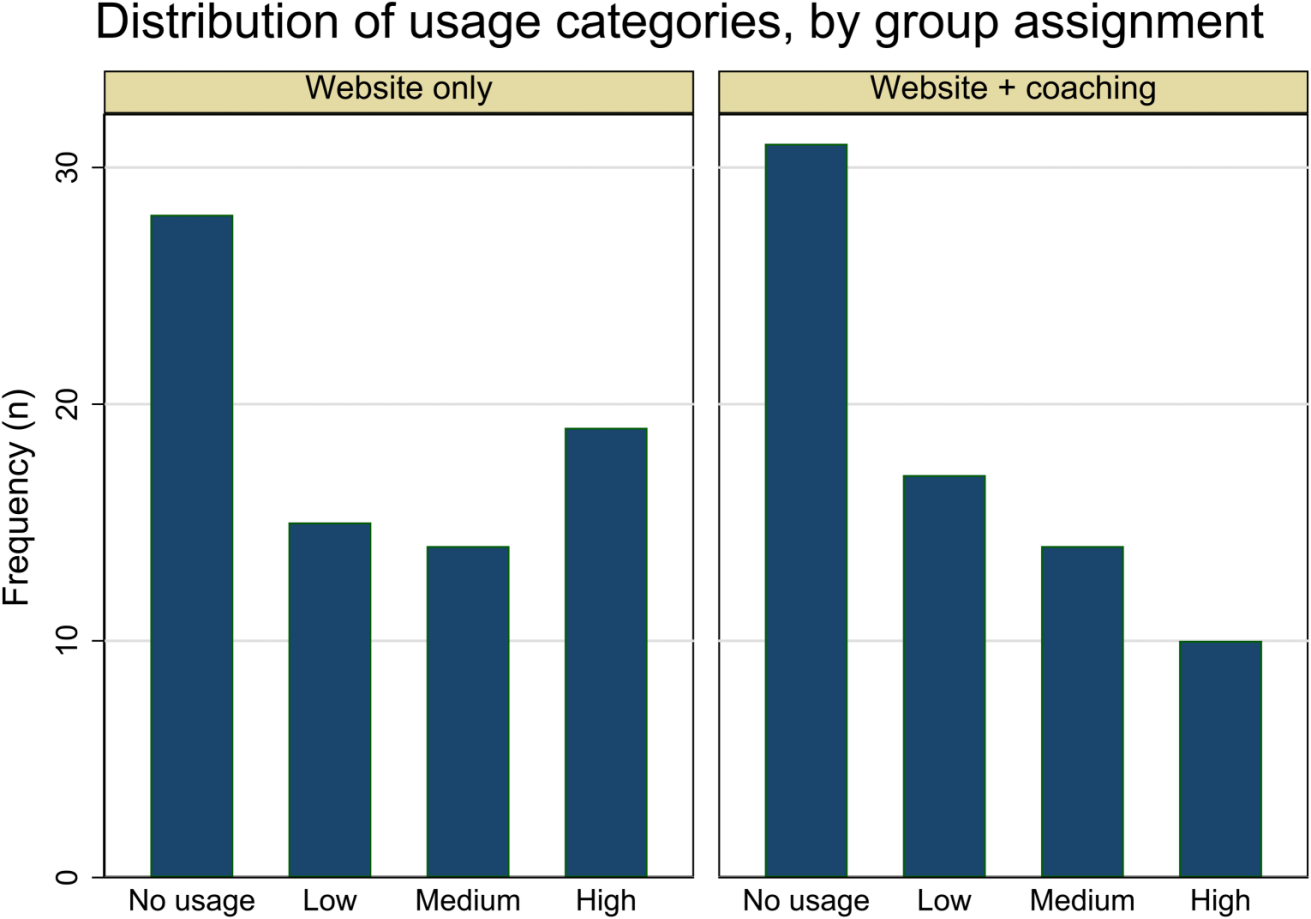
Distribution of usage categories by group assignment.

Treatment group assignment did not appear to influence the likelihood of logging into the website (Table 2, RR: 0.93, 95% CI: 0.72, 1.19), nor did it have a meaningful impact on the categorical extent of web use (Table 3; categorical findings are expressed on the probability scale). Categorical findings suggested that the probability of being in any of the four web use groups (no, low, medium, or high usage) was statistically indistinguishable between intervention arms, although the distribution differed slightly by treatment group (Figure 1). The difference in the probability of logging in vs. not logging in, comparing the web + coaching and web-only intervention groups, was also non-significant (RD: −0.05, 95% CI: −0.20, 0.11). Taken together, these analyses suggest that, aside from gender, no other MS caregiver characteristic was consistently linked to the probability of logging into the website.

**Table 2.** Likelihood of logging in (vs. not logging in), by treatment assignment.

	**RR^a^**	**CI^b^**	**RD^c^**	**CI^b^**
Group assignment (2 v. 1)	0.93	0.73, 1.19	−0.05	−0.20, 0.11

^a^ Risk ratio.

^b^ Confidence interval.

^c^ Risk difference.

**Table 3.** Categorical web use, by treatment assignment.^a^

	**No usage**	**Low**	**Medium**	**High**
Base probability^b^	**0**.**4**	**0**.**22**	**0**.**19**	**0**.**2**
Change associated with group assignment: 2 v. 1^c^	0.1	−0.001	−0.03	−0.06
P-value	0.18	0.86	0.19	0.18

^a^ Based on an ordinal logistic regression model (probabilities obtained with Stata's mtable command). ^b^Probability of categorical web use among people in group 1 (web only). ^c^Difference in base probability associated with being in the experimental group (web + counseling group).

**Figure 2.**
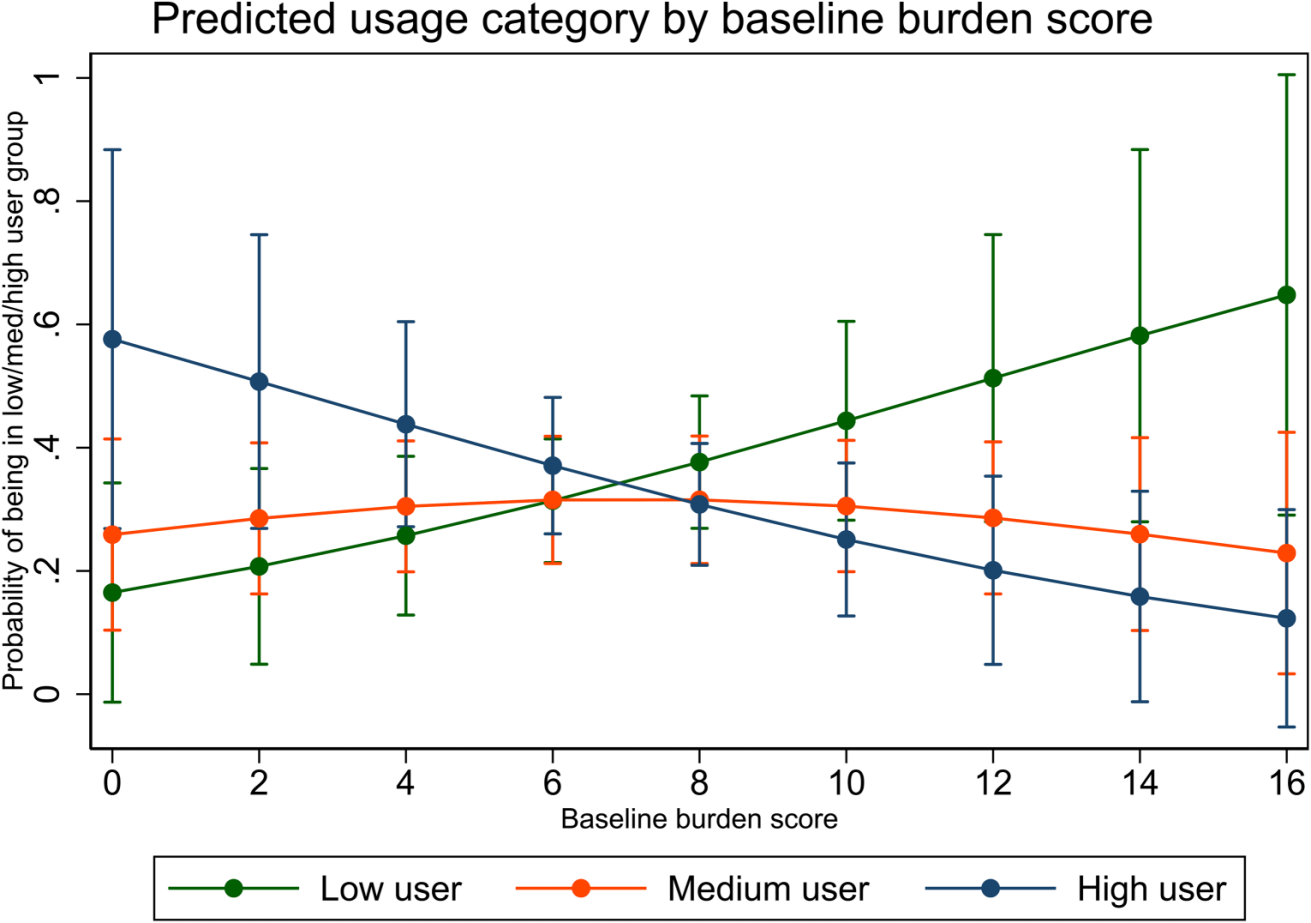
Predicted usage category by baseline burden score.

**Factors associated with categorical (low, medium, high usage) web use.** We next assessed whether characteristics *beyond* intervention assignment influenced categorical web use restricting the analysis to participants who had logged into the website (Appendix 1). Approximately one third of the overall sample who did not access the website at all, and five participants who logged in but did not navigate beyond the homepage (“no usage” category) were excluded, resulting in 89 caregivers who were classified as “users”. While none of the coefficients were statistically significant at conventional levels, we observed a substantively interesting association between usage category and both age (POR: .96, 95% CI: .91, 1.00) and baseline burden (POR: .85, 95% CI: .69, 1.03). Although both confidence intervals contain the null, the range and position of the intervals suggests that older users and users experiencing higher levels of baseline burden may be less engaged users of the website; see Figure 2, which plots (model-derived) predicted probabilities of being in each usage category based on participants’ baseline burden score. This plot suggests that MS Caregivers with low baseline burden scores are more likely to be in the “high user” category, whereas MS Caregivers with high baseline burden scores are more likely to be in the “low user” category (although CIs overlap).

### RQ2. Is there a relationship between caregivers’ baseline burden/depression/anxiety/stress measures and the website pages they access?

The previous analyses demonstrated the extent to which caregivers accessed the website. They do not, however, illustrate the nature of MS Caregivers’ engagement with specific topic/content areas. Table 4 tabulates the number and percentage of users (n = 89 who accessed website) “clicking on” each of the six topics. Differences by treatment group were tested to determine whether those who accessed each topic were more likely to be allocated to the website-only or website + coaching group (see Table 4; graphical illustration in Appendix 2). As is evident from both the table and the plot, Topic 3 (*Caring for a loved one*) and Topic 5 (*Caring for yourself*) were accessed by the greatest number of participants. Topic 4 (*MS and COVID-19*) was the least-accessed module. However, access patterns differed by treatment group: significantly more caregivers in the website-only group accessed Topic 5 (*Caring for yourself*; 61.67% compared to 38.33%: p = .04). Another compelling (but not statistically significant) finding was seen for Topic 3; 60.3% of caregivers in the website only group accessed this topic compared to 37.7%; p = .06 in the website + coaching group. This suggests that users who didn’t receive coaching (group 1) may have relied more heavily on the website as a resource.

**Table 4.** Topic access by treatment group assignment.

	**Total accessed^a^**	**Group 1 (web only)^b^**	**Group 2 (web + coaching)^b^**	***p*-value^c^**
Topic 1: Information about MS	43 (48.31%)	23 (53.49%)	20 (46.07%)	0.94
Topic 2: Obtaining reliable information about Multiple Sclerosis	37 (41.57%)	21 (56.76%)	16 (43.24%)	0.65
Topic 3: Caring for your loved one with Multiple Sclerosis	63 (70.79%)	38 (60.32%)	25 (39.68%)	0.06
Topic 4: COVID-19 and MS	32 (35.96%)	16 (50.00%)	16 (50.00%)	0.58
Topic 5: Caring for yourself	60 (67.42%)	37 (61.67%)	23 (38.33%)	0.04
Topic 6: Planning and decision-making	49 (55.06%)	30 (61.22%)	19 (38.78%)	0.13

^a^ Out of 89 people who did (versus did not) access the website.

^b^ Percentages reflect the share of total access by treatment group (for example, 53.5% of the 43 people who accessed Topic 1 were from Treatment group 1).

^c^ Based on chi2 tests for each topic/module.

Web usage was also measured by the percentage of content accessed by topic (0–100%). Those who accessed the website engaged with an average of 10% of the available content in most modules/topics (but closer to 20% of the contents of Topic 3). To determine whether depth of engagement varied by baseline scores, we used t-tests to compare percentage of topic viewed among users with high baseline scores (defined as above the clinical threshold of 8 on the Zarit Caregiver Burden Scale,^32^ and >75th percentile for depression, anxiety, stress, and distress) to MS Caregivers below these thresholds. While baseline measures of depression, anxiety, stress, and distress did not appear to be related to the extent of engagement with specific web topics (Appendix 3), caregiver burden again emerged as a potentially important factor. Users with baseline burden scores above the clinical threshold of 8 accessed significantly less of Topic 1 compared to users with low/moderate baseline burden (Table 5). This trend, though not statistically significant, was also seen for other topics.

**Table 5.** Average engagement by topic/web module among users (percentage of topic accessed): normal vs. high baseline burden.

Baseline score	≤8	>8	*p*-value
Topic 1: Information about MS	13%	6%	0.02
Topic 2: Obtaining reliable information about MS	13%	6%	0.08
Topic 3: Caring for your loved one with MS	21%	15%	0.15
Topic 4: COVID-19 and MS	11%	7%	0.24
Topic 5: Caring for yourself	12%	8%	0.10
Topic 6: Planning and decision-making	12%	6%	0.06

### RQ3. Does website use influence outcomes within and between groups (website-only and website + coaching)?

**Web use and mental health post intervention.** The relationship between web usage and outcome measures post-intervention was assessed using regression models (Table 6); findings illustrate the change in outcome measures at t2 associated with a one-unit shift in the applicable predictor variable, conditional on the other model covariates. These models help estimate, for example, whether high levels of web use were associated with a reduction in depression (or other endpoints) compared to low use, conditional on model covariates. Caregiver burden was only measured at baseline, so we were not able to assess the relationship between web usage and burden.

**Table 6.** Categorical web usage and mental health measures at T2 (n = 133).

	**Depression at t2**	**Anxiety at t2**	**Stress at t2**	**Distress at t2**
Usage cat (ref = Nonusers)				
Low	1.34 (0.15, 2.53)	0.80 (−0.15, 1.75)	1.08 (−0.19, 2.34)	0.37 (−0.09, 0.82)
Med	0.17 (−0.79, 1.13)	0.11 (−0.51, 0.74)	−0.14 (−1.30, 1.03)	0.12 (−0.37, 0.61)
High	−0.06 (−0.92, 0.81)	0.53 (−0.09, 1.16)	−0.11 (−1.28, 1.06)	0.13 (−0.38, 0.64)
Group assign = 2	0.30 (−0.46, 1.07)	0.44 (−0.09, 0.97)	1.09 (0.18, 2.01)	0.28 (−0.09, 0.65)
Outcome measure@ t1^a^	0.87 (0.81, 0.94)	0.89 (0.82, 0.96)	0.83 (0.77, 0.88)	0.76 (0.69, 0.84)
CP female	−1.25 (−2.02, −0.48)	−0.60 (−1.15, −0.05)	−0.97 (−1.83, −0.11)	−0.19 (−0.55, 0.16)
Time since diagnosis	0.01 (−0.03, 0.04)	−0.01 (−0.04, 0.02)	0.002 (−0.04, 0.05)	−0.01 (−0.03, 0.003)
CP hours per week (vs. < 10)				
10–20	−1.67 (−2.74, −0.61)	−0.54 (−1.25, 0.16)	−1.51 (−2.69, −0.32)	−0.43 (−0.95, 0.08)
21–30	−0.34 (−1.62, 0.95)	−0.07 (−1.19, 1.05)	0.23 (−1.59, 2.06)	−0.25 (−0.89, 0.40)
31–40	−1.96 (−3.08, −0.84)	0.13 (−0.89, 1.15)	−1.13 (−3.11, 0.85)	−0.47 (−1.08, 0.14)
>40	−0.25 (−1.17, 0.66)	0.41 (−0.27, 1.09)	0.47 (−0.55, 1.50)	0.20 (−0.26, 0.66)

^a^ This measure changes by model specification - for example, it's the baseline depression coefficient in the depression model, it's the baseline anxiety coefficient in the anxiety outcome model, etc.

As anticipated, baseline values on outcome measures were consistently predictive of measures at follow-up. Female gender was associated with lower (better) scores post intervention for nearly all endpoints (conditional on covariates)—for example, follow-up (t2) depression scores among female caregivers were 1.25 points lower (95% CI: −2.02, −0.48) than male caregivers’ scores on average. Categorically, hours of care provided were also associated with lower post interventions scores, but there were no clear trends/gradients in this relationship. In the basic models, web use did not appear to be consistently associated with DASS measures at follow-up; however, we did observe some evidence of an interaction between treatment group and usage category (Appendix 4), with reduced distress among “medium” users in the website + coaching group, but increased anxiety among “low” users in the website + coaching group. Aside from these findings, results from the models containing the usage category*group assignment interaction term were generally consistent with our initial findings.

## Discussion

This analysis sought to better understand website usage patterns, and to identify any detectable shifts in mental health measures plausibly associated with usage overall and by treatment group. Key findings point to three important areas for service providers and policymakers: 1) female MS Caregivers’ access, use and benefit from curated websites differentially than males; 2) MS Caregivers experiencing high levels of caregiver burden may be less likely to access and be engaged users (although these findings were not statistically significant), and 3) usage patterns differ based on access to non-website intervention components.

Regardless of health condition and/or disability, most caregivers are female, with estimates ranging from 53 to 68% of family and informal caregivers (American Psychological Association www.apa.org/pi/about/publications/caregivers/faq/statistics#). Participants in caregiver studies are often even higher, with 80% or more female.^36,37^ Of the 148 participants in this study, 54% of participants reported being female, with a high proportion also identifying as partners/spouses (as opposed to friends or other family members). Given that the incidence of MS is 3:1 in favor of women,^38^ this represents disproportionately higher participation by female than male MS Caregivers. Interestingly, this also mirrors participation in many studies of MS Caregivers, which report female participation rates of 67% or greater.^18,39–41^ Two recent studies contradict this trend. In a study recruiting dyads for a resilience building intervention, proportions of people with MS (80% female) and caregivers (32% female) more closely approximated incidence and prevalence rates.^17^ Petrikis also reported lower proportions with only 46% female participants.^39^

In addition to being more likely to participate, female MS Caregivers in this study were significantly more likely to access the website than males and had better outcomes (i.e., experienced less depression, anxiety, and stress) post intervention, regardless of group assignment. Together, these findings suggest differential responses by, and benefits for female, compared to male MS Caregivers. It is possible that women are not only more likely to be caregivers but may also experience caregiving differently than males. Examining the relation between caregiver quality of life and Parkinson's disease symptoms, Henry et al.^42^ found that female caregivers experienced more strain and poorer self-care than male caregivers. Kazemi et al.^43^ found that male and female caregivers of people following stroke used different coping strategies. Regardless of the underlying explanation, these findings point to differential needs of caregivers. Research to further discern the specific needs and interests of female vs. male MS Caregivers may be of value in curating content for future websites.

A somewhat concerning finding was that MS Caregivers experiencing high levels of caregiver burden were less likely to be heavily engaged users. In other words, caregivers already overwhelmed by caregiving were less likely to take advantage of available resources. Evidence was two-fold. Caregivers with high burden scores engaged in significantly less Topic 1 content, with a similar trend for all other topics. Figure 2 confirms this, showing that caregivers with low baseline burden scores were less likely to be in the high use category. This finding is not universal. Halstead attributed MS dyad drop out to medically related issues and family bereavement.^17^ MS caregivers in a second study^18^ reported the intervention took much time given their caregiver duties. However, Martindale-Adams et al.^40^ found that MS Caregivers with higher baseline burden scores on the Zarit Burden Interview (12-item) were more likely to complete all six sessions of the intervention. Future research is needed to investigate the concern that caregivers most in need are the least likely to be able to find time and energy to access available resources. In the meantime, health and social care providers can have the most confidence referring females with mild to moderate levels of self-reported burden to curated websites.

Finally, our findings suggest that in the absence of coaching, websites alone may provide a useful, low-cost option for some caregivers. While not all caregivers will use these tools (access rates were relatively modest overall in our study), two topics stood out as most accessed—Topic 3, caring for a loved one, and Topic 5, caring for yourself. However, caregivers in the website + coaching group did not access these topics in the same depth as those in the website-only group, suggesting that access to coaching reduced or replaced engagement with the curated website. Few comparative studies could be found, though one feasibility study with a similar design has been reported^18^ and helps to deepen understanding. A self-help group (receiving written material weekly) was compared to both an enhanced self-help (written material plus weekly phone call) and a no-intervention control. Results demonstrated within group improvements for the enhanced self-help group but not the self-help or control group. Feedback on the written material found the scientific language, lack of first voice content and time to read to be difficult for caregivers, leading researchers to re-think the intervention content prior to further study. Although evidence is emerging, further work is needed to fully understand whether curated websites should be considered stand alone, replacement or enhancements to caregiver interventions. It will also be important to better understand how to optimize the interface and content of curated websites to maximize user engagement.

Although our findings shed light on how best to support an under-researched population of caregivers, this work is not without limitations. First, given our relatively small sample, some of our analyses may have been underpowered; however, the randomized nature of the original RCT guards against several biases. Second, the sociodemographic distribution of our caregivers (specifically the gender distribution) was not what we expected: as the majority of MS patients are female, we anticipated to see more male caregivers (e.g., husbands/partners) in the sample. This may impact the generalizability of our findings. We also made the choice to exclude one person in the sample who reported a gender identity of “Other” rather than arbitrarily assign them to one of the other groups. Finally, our findings may be enriched by incorporating a qualitative lens (e.g., participant interviews) in future studies. This would be particularly helpful in determining web use barriers/facilitators and to further understand the low rates and depth of access.

## Conclusions

Our findings provide insight into who accesses and benefits from curated websites. Knowing that females with low to medium levels of caregiver burden are most likely to access and benefit from a curated website will help clinical decision-making and tailoring of interventions. By contrast, our findings suggest male caregivers and those experiencing high levels of burden may be less likely to access these resources, suggesting the need for alternative intervention pathways. Future research to investigate issues of time, complexity, and content of websites vs. the impact of relational support from in-person or virtual care provider is needed to inform the design of virtual care interventions. Future research could also investigate whether early access to information and/or coaching can prevent or delay caregiver burden.

## Supplemental Material

sj-pdf-1-dhj-10.1177_20552076241228403 - Supplemental material for Factors influencing how informal caregivers of people with multiple sclerosis access and use a curated intervention website: Analysis from an RCT Click here for additional data file. Supplemental material, sj-pdf-1-dhj-10.1177_20552076241228403 for Factors influencing how informal caregivers of people with multiple sclerosis access and use a curated intervention website: Analysis from an RCT by Tanya Packer, Nichole Austin, Michelle Lehman, Sara L Douglas and Matthew Plow in DIGITAL HEALTH

sj-docx-2-dhj-10.1177_20552076241228403 - Supplemental material for Factors influencing how informal caregivers of people with multiple sclerosis access and use a curated intervention website: Analysis from an RCT Click here for additional data file. Supplemental material, sj-docx-2-dhj-10.1177_20552076241228403 for Factors influencing how informal caregivers of people with multiple sclerosis access and use a curated intervention website: Analysis from an RCT by Tanya Packer, Nichole Austin, Michelle Lehman, Sara L Douglas and Matthew Plow in DIGITAL HEALTH

## Appendix

### Appendix 1: caregiver characteristics and categorical web use

Model notes:

The validity of ordinal regression models rests upon the proportional odds assumption; we verified that this assumption was met using Stata's omodel command (p = 0.39, where a p-value of > .05 suggests proportionality of odds across response categories). Estimates are expressed as proportional odds ratios (PORs) and interpreted as the odds of being in a higher (vs. lower) usage category given a 1-unit increase in the predictor, holding other covariates constant (we assume these odds are consistent/proportional across all levels of the outcome variable).

We dichotomized certain categorical variables (e.g., income, employment) in the interest of improving model fit; the levels are described in the footnotes. We excluded from this analysis any MS caregiver in the “no usage” category, including those who never navigated past the homepage (n = 5).

**Appendix Table 1.** Categorical web usage (proportional odds ratio^a^ (95% CI)).

	POR	95% CI
Female gender (v. male)	0.87	0.33, 2.28
Age	0.96	0.91, 1.00
White (v. other)	1.28	0.35, 4.76
Spouse/partner (vs. other)	1.22	0.28, 5.24
Employed^b^ (vs. not employed/retired)	0.69	0.21, 2.25
Income >=$50,000^c^	1.47	0.44, 4.91
Rural (binary)	0.61	0.20, 1.82
DASS: Depression	0.95	0.84, 1.07
DASS: Anx	0.95	0.83, 1.09
DASS: Stress	1.08	0.96, 1.20
CP burden	0.85	0.69, 1.03
CP distress	1.09	0.81, 1.47
20 + caregiving hours per week^d^	0.91	0.32, 2.55
Progressive MS (vs. relapsing)	0.94	0.34, 2.56
Time since diagnosis (years)	1.02	0.97, 1.07
Group assignment (2 vs. 1)	0.53	0.20, 1.39

^a^ Estimates reflect the odds ratio of being in a higher usage group (e.g., “High” vs. “Low"/"Medium”, or “High"/"Medium” vs. “Low”) with a 1-unit change in the applicable predictor, holding other factors constant.

^b^ Part or full-time.

^c^ vs. <$50k.

^d^ vs. < 20 h/week.

#### Appendix 2: Topic access by treatment group assignment (frequencies/counts)

See Online Appendix Figure 1 JPEG file.

**Appendix Table 2.** Average engagement by topic/web module among users (percentage of topic accessed), by baseline score.

Baseline Percentile	≤75th	>75th	*p*-value
**Depression**			
Topic 1	12%	7%	0.19
Topic 2	11%	7%	0.31
Topic 3	21%	14%	0.15
Topic 4	10%	8%	0.62
Topic 5	11%	9%	0.59
Topic 6	11%	8%	0.50
**Anxiety**			
Topic 1	11%	11%	0.98
Topic 2	11%	9%	0.63
Topic 3	20%	11%	0.10
Topic 4	10%	10%	0.98
Topic 5	11%	8%	0.46
Topic 6	10%	10%	0.94
**Stress**			
Topic 1	11%	9%	0.50
Topic 2	11%	7%	0.29
Topic 3	19%	18%	0.80
Topic 4	9%	11%	0.58
Topic 5	11%	10%	0.68
Topic 6	11%	9%	0.55
**Distress**			
Topic 1	11%	10%	0.86
Topic 2	12%	5%	0.15
Topic 3	19%	17%	0.66
Topic 4	10%	7%	0.37
Topic 5	11%	9%	0.64
Topic 6	10%	10%	0.95

#### Appendix 4: Web use and mental health measures at follow-up (t2), with interaction

**Appendix Table 3.** Categorical web usage and mental health measures at T2 (incl. interaction term).

	**Depression at t2**	**Anxiety at t2**	**Stress at t2**	**Distress at t2**
Usage cat (ref = Nonusers)				
Low	0.32 (−1.15, 1.79)	−0.30 (−1.52, 0.92)	0.42 (−1.62, 2.45)	0.29 (−0.45, 1.03)
Med	0.75 (−0.57, 2.07)	0.19 (−0.71, 1.09)	0.52 (−1.35, 2.38)	0.85 (0.08, 1.62)
High	−0.35 (−1.43, 0.73)	0.20 (−0.57, 0.97)	−0.92 (−2.35, 0.50)	0.07 (−0.52, 0.65)
Group assign = 2	−0.003 (−1.28, 1.28)	−0.09 (‘−0.76, 0.58)	0.66 (−0.77, 2.10)	0.48 (−0.01, 0.98)
Usage cat*group assign.				
Low*group 2	1.85 (−0.30, 4.01)	2.00 (0.26, 3.73)	1.21 (−1.23, 3.65)	0.13 (−0.82, 1.09)
Med*group 2	−1.14 (−3.01, 0.72)	−0.21 (−1.39, 0.96)	−1.28 (−3.54, 0.97)	−1.39 (−2.29, −0.48)
High*group 2	0.71 (−1.21, 2.62)	0.63 (−0.63, 1.89)	2.24 (−0.30, 4.78)	0.32 (−0.79, 1.43)
Outcome measure@ t1^a^	0.87 (0.81, 0.93)	0.88 (0.82, 0.95)	0.83 (0.78, 0.88)	0.77 (0.70, 0.84)
CP female	−1.17 (−1.93, −0.42)	−0.55 (−1.10, −0.003)	0.93 (−1.79, −0.07)	0.16 (−0.51, 0.18)
Time since diagnosis	0.01 (−0.03, 0.04)	−0.01 (−0.04, 0.02)	0.003 (−0.04, 0.05)	−0.01 (−0.03, 0.003)
CP hours per week (vs. < 10)				
10–20	−1.69 (−2.70, −0.69)	−0.54 (−1.22, 0.14)	1.56 (−2.68, −0.43)	−0.45 (−0.97, 0.07)
21–30	−0.25 (−1.51, 1.01)	0.07 (−1.09, 1.23)	0.32 (−1.44, 2.07)	−0.26 (−0.82, 0.30)
31–40	−1.96 (−3.12, −0.79)	0.16 (−0.81, 1.14)	−1.21 (−3.22, 0.81)	−0.51 (−1.17, 0.14)
>40	−0.27 (−1.16, 0.62)	0.43 (−0.23, 1.09)	0.40 (−0.59, 1.40)	0.17 (−0.23, 0.58)

^a^ This measure changes by model specification - for example, it's the baseline depression coefficient in the depression model, it's the baseline anxiety coefficient in the anxiety outcome model, etc.
