# Supplementary figures and images for: Factors influencing how informal caregivers of people with multiple sclerosis access and use a curated intervention website: Analysis from an RCT
Source: Digit Health. 2024 Feb 8;10:20552076241228403. doi: 10.1177/20552076241228403 (PMC10858664; doi:10.1177/20552076241228403)

# Module access, by group assignment

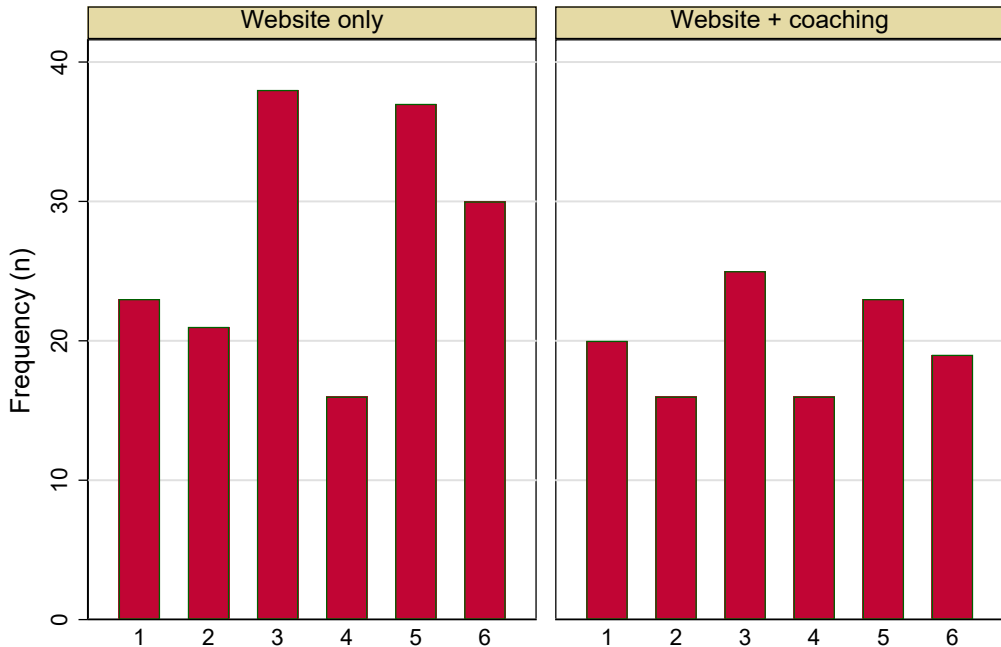

Supplement: sj-pdf-1-dhj-10.1177_20552076241228403 - Supplemental material for Factors influencing how informal caregivers of people with multiple sclerosis access and use a curated intervention website: Analysis from an RCT [file sj-pdf-1-dhj-10.1177_20552076241228403.pdf]
